# From Implantation to Birth: Insight into Molecular Melatonin Functions

**DOI:** 10.3390/ijms19092802

**Published:** 2018-09-17

**Authors:** Gianfranco Carlomagno, Mirko Minini, Marco Tilotta, Vittorio Unfer

**Affiliations:** 1 Department of R&D, Lolipharma Srl, 00156 Rome, Italy; m.tilotta@lolipharma.it; 2 Department of Experimental Medicine, Sapienza University of Rome, 00185 Rome, Italy; minini.1699603@studenti.uniroma1.it; 3 Department of Developmental and Social Psychology, Faculty of Medicine and Psychology, Sapienza University of Rome, 00185 Rome, Italy; vunfer@gmail.com

**Keywords:** melatonin, pregnancy, oocyte quality, embryo implantation, fetal development

## Abstract

Melatonin is a lipophilic hormone synthesized and secreted mainly in the pineal gland, acting as a neuroendocrine transducer of photoperiodic information during the night. In addition to this activity, melatonin has shown an antioxidant function and a key role as regulator of physiological processes related to human reproduction. Melatonin is involved in the normal outcome of pregnancy, beginning with the oocyte quality, continuing with embryo implantation, and finishing with fetal development and parturition. Melatonin has been shown to act directly on several reproductive events, including folliculogenesis, oocyte maturation, and corpus luteum (CL) formation. The molecular mechanism of action has been investigated through several studies which provide solid evidence on the connections between maternal melatonin secretion and embryonic and fetal development. Melatonin administration, reducing oxidative stress and directly acting on its membrane receptors, melatonin thyroid hormone receptors (MT1 and MT2), displays effects on the earliest phases of pregnancy and during the whole gestational period. In addition, considering the reported positive effects on the outcomes of compromised pregnancies, melatonin supplementation should be considered as an important tool for supporting fetal development, opening new opportunities for the management of several reproductive and gestational pathologies.

## 1. Introduction

Melatonin is a lipophilic hormone synthesized and secreted mainly in the pineal gland, acting as a neuroendocrine transducer of circadian and circannual photoperiodic [1]. It is released cyclically during over a 24-h period, with a peak during the night [2]. Moreover, melatonin is a potent antioxidant, with greater efficacy than vitamin C and E [3]. In fact, together with its metabolites, this hormone directly removes reactive oxygen species (ROS) [4], increasing the gene expression of several antioxidant enzymes, like superoxide dismutase (SOD) and glutathione (GSH), inhibiting pro-oxidative enzymes, and reducing cellular oxidative damage. Melatonin is synthesized in high concentrations in the mitochondria, constituting one of the most important factors for ROS elimination [5].

In addition to having antioxidant function, melatonin plays a key role in several physiological functions, such as in the circadian rhythm [6].

The circadian rhythm is present in almost all the organisms, and organizes the physiological systems in order of time, aligning them with the 24-h environmental cycles. In fact, it is involved in a series of physiological processes, such as sleep/wake [7], physique temperature regulation [8], and hormone secretion [9]. In mammals, circadian rhythm regulation is based on a series of feedbacks between groups of genes known as “clock genes”, expressed in almost all tissue types [10]. Within the circadian rhythm, melatonin participates through the regulation of critical gene expressions [11].

Melatonin is also synthetized in other tissues, such as placental tissue, retina, brain, bone marrow, and lymphocytes, thereby regulating local physiological processes [12].

Several studies have displayed how the interruption of the circadian rhythm through night-time work, which causes exposure alteration to light, negatively affects the implantation and pregnancy success at a molecular level, triggering an increase in infertility, menstrual deregulation, and miscarriages [13]. In fact, melatonin sends photoperiodic information that regulates reproductive activity, improving ovarian functions and participating in the follicular development process, including ovulation [14].

Furthermore, being able to rapidly pass through the placenta, melatonin sends photoperiodic information to the fetus, supporting tissue differentiation and hormonal metabolism [15].

From a clinical point of view, it must be borne in mind that endogenous levels of melatonin are reduced with increasing age; this event correlates with cellular senescence, due to the exposition of cells to ROS activity [16]. Aging negatively affects female fertility by lowering oocyte quality, with the increase of aneuploidy [17], and by reducing the follicle and oocyte pool [18]. The main cause of ovarian aging seems to be oxidative stress [19]. So, melatonin exogenous supplementation may also be useful as a treatment for women who are near the end of their reproductive age and want to become pregnant.

In general terms, reproductive disorders triggered through hormonal alterations represent one of the main causes of infertility [20]; this is why many studies have been focused on understanding melatonin functions during pregnancy [21,22].

## 2. Molecular Mechanisms of Embryo Implantation

The embryonic implantation is a key and complex process that requires correct communication between blastocyst and uterus. The establishment of uterine receptivity, preimplantation embryo development, and embryo implantation events are mainly regulated by cytokines, chemokines, growth factors, and steroid hormones. Steroid hormones, especially estrogen and progesterone (P4), play important roles in supporting endometrial preparations to establish endometrial receptivity, and allow the maternal clock to synchronize with the embryonic one [23].

In fact, after ovulation, progesterone production in the ovary allows endometrial differentiation, favoring a receptivity state for the implant [24]. About 24 h after fertilization, the embryo begins cell divisions from which blastomeres derive, increasing the total cell number but not the total volume, thanks to a compaction process. During this phase, the embryo is pushed by oviduct cilia towards the uterus, and the internal cells acquire a defined polarity, while the external cells remain apolar and starts to dwindle. From this differentiation process, the blastocyst is derived; it is characterized by an internal cell mass (ICM) and trophectoderm (TE). In the uterus, the blastocyst remains adherent, and for 48 h increases its dimensions. The TE cells, positioned at the external limit of the embryo, are responsible for the endometrial connection. Subsequently, TE cells secrete enzymes to penetrate the endometrium, starting syncytiotrophoblast formation. Thereafter, trophectoderm forms several embryonic structures from which the placenta will derive. With regard to the development of the fetus, ICM cells will be responsible of all embryonic and fetal tissues [25].

**Figure 1:**
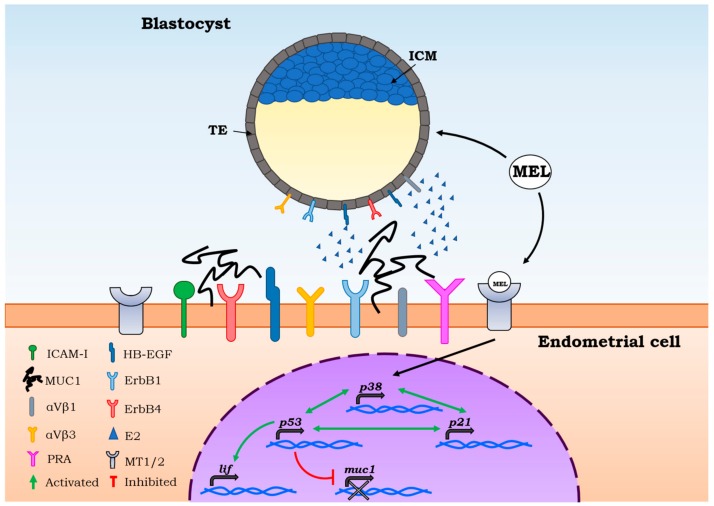
Pre-implantation stage. Physiological secretion of melatonin allows it to interact with melatonin membrane receptors (MT1-MT2) in endometrial cells and the blastocyst. Melatonin signaling creates a positive feedback loop among *p53*, *p38,* and *p21*, activating *lif* transcription and inhibiting mucin 1 secretion. The result is a better interaction among the adhesion proteins present at the membrane level on endometrial cells and the blastocyst.

In vivo studies in mice [26] have identified a probable embryo receptivity window of about 4 days, which occurs about 7–11 days after the luteinizing hormone (LH) peak. One of the main estrogen mediators responsible for implantation seems to be leukemia inhibitory factor (LIF), an interleukin-6 family member, regulated by p53 [27], that guarantees the uterine receptivity in the implant (Figure 1). Indeed, scientific studies have reported implant failure in mice when this factor was suppressed.

In molecular terms, endometrial receptivity is an accurate process involving the acquisition of adhesion ligands and receptors. Among the actors involved in this series of events is αVβ3, an integrin present and up-regulated on the apical surface of luminal endometrial cells during implantation, even if its embryonic ligand still hasn’t been detected [28]. Another molecule responsible for the regulation of this process is mucin (MUC1), which is present on the endometrial cell surface and is characterized by anti-adhesive properties [29]. Considering the MUC1 activity relative to implantation, it is clear that a local action is needed to remove this barrier, as reported in some studies, where MUC1 down-regulation in endometrial cells was observed in the embryo paracrine action in rabbits [30] (Figure 1).

The existence of conflicting mechanisms allows us to set the timing of the implantation process. Thanks to these molecular events, implantation can be divided into three finely regulated stages: apposition, adhesion, and penetration. These three phases are completed in the “implant window”, a period in which the endometrium is receptive to the blastocyst [31].

The pre-implantation stage, known as apposition, was observed in rodent studies [32]. During apposition, TE cells are in close adherence with the endometrial epithelial wall [33]. This close apposition would seem to support the correct embryonic poles orientation, which is critical for placental development [32]. At the molecular level, the pre-implantation stage has been described with the identification of different adhesion molecules, such as αVβ1 integrins and the intracellular adhesion molecule (ICAM-I) [34] (Figure 1).

After this phase, the trophoblast and the luminal epithelium establish a strong bond (adhesion). Embryonic adhesion would seem to be modulated by microenvironmental signals which cause up-regulation in adhesion proteins [35]. Once adhesion has occurred, the blastocyst is inserted into epithelial tissue, and switches into stroma to connect to the maternal vascular system [36]. During this phase, the blastocyst cells begin to activate the matrix metalloproteinase family members, which allow proteolytic digestion of the maternal stromal extracellular matrix, favoring penetration and implantation.

In recent years, the identification of the implantation and development mechanisms has allowed us to understand this process, improving also in vitro fertilization techniques. Despite this, so far only 10–15% of embryos implanted after in vitro fertilization have a positive outcome [37]. To clarify these data, it is important to highlight what factors are involved. Among these, ROS overproduction is, undoubtedly, one of the main factors, being responsible for embryonic damage [38] through the induction of apoptosis [39].

## 3. Melatonin Functions on Oocyte Quality and Embryo Implantation

The role of melatonin in human reproduction has been deeply investigated through direct actions on the ovary, supporting the development of good quality oocytes and embryos. In fact, when injected in a systemic way, melatonin was found in higher concentrations in ovary tissue, confirming a targeted action. From the pineal gland, by circadian cyclical release, melatonin mediates the down-regulation of gonadotropin-releasing hormone (GnRH) in a cyclic time [40], assuming a central role in the reproductive system.

Normally, melatonin levels positively correlate with follicular growth, assuming an important role in the ovulatory phase [41]. Melatonin plays an important role as a regulator of several phases of ovulation, as demonstrated by the presence of melatonin binding sites in the membrane fraction of human granulosa cells (GCs), and of melatonin membrane receptors (MT1 and MT2) in granulosa (GC)/luteal cells. In this site, melatonin acts like natural guardian of primordial follicle pool. Its protective action doesn’t allow phosphorylation of several important molecular factors. In their work, Jang et al. display how melatonin blocks the activation of extracellular signal-regulated kinase (ERK), protein kinase B (Akt), phosphatidylinositol (3,4,5)-trisphosphate (PIP3), glycogen synthase kinase 3 beta (GSK3β), Forkhead box O3 (FOXO3a), and phosphatase and tensin homolog (PTEN) [42]. The phosphorylation block of PTEN inhibits the PI3K pathway, as well as Akt activation and its down-stream effectors (ERK, GSK3β, FOXO3a) [43].

Another actor in the implantation process is the immune system. During follicle rupture, immune system cells release prostaglandins and cytokines to increase the capillary permeability. This important process leads to an elevated production of free radicals and ROS [44].

High levels of melatonin were found in human pre-ovulatory follicular fluid [45], reducing oxidative stress in the follicles and protecting the oocytes from damage caused by free radicals [46]. Three-fold higher levels of melatonin were found in human preovulatory follicular fluid (FF) than serum levels. Moreover, melatonin concentrations were higher in the fluid of large follicles than in the fluid of small follicles in patients undergoing in vitro fertilization (IVF)-embryo transfer, confirming its fundamental role in this context. In follicular fluid, melatonin does not occur exclusively from its uptake from blood, but could be synthetized by ovarian cells, such as cumulus cells [47]. In this way, melatonin functions can be improved in the ovaries, leading to local specific actions. This melatonin supplement is necessary to reduce oxidative stress in follicles triggered by inflammation-like processes.

Another important data is the mechanism of selection of the ovulatory follicle that seems to be linked with the mRNA expression encoding LH receptors in GCs. In this regard, melatonin treatment increases the mRNA expression of LH receptors in human GCs, highlighting the important role of this molecule in the female fertility. Moreover, melatonin can influence sex steroid production at different phases of ovarian follicular maturation, increasing progesterone production in preantral follicles, as demonstrated in mice by Adriaens et al. [48]

Furthermore, an interesting hypothesis views melatonin as an important factor in the seasonal variability of human fertility. Indeed, for all human populations, a seasonality in birth rapports was reported [49]; this variability in fertilization rates, and in the embryo quality, might depend on changes in melatonin secretion, as suggested by some studies [50].

In this regard, the in vitro use of melatonin on embryo culture has been shown to cause a reduction of oxidative stress and apoptosis [51]. Indeed, scientific studies have reported that melatonin is able to increase the number of blastocyst cells, increase GSH levels, and reduce oxidative stress and apoptosis [52], having a protective effect on embryos.

These effects may also derive from actions on the oocyte itself: the early stages of embryogenesis are specifically subject to information contained at the oocyte level. High quality oocytes give rise to well-developed embryos. In particular, during the second meiotic division, the oocyte accumulates oxidative stress, which must be reduced to obtain a good quality embryo [53].

In addition to the direct antioxidant activity, melatonin-induced functions are directly mediated by binding to melatonin membrane receptors MT1 and MT2 [54]. Following stimulation, melatonin receptors activate several signaling pathways involved in embryo implantation: the MT1, MT2, p53, and LIF levels in the uterus were evaluated in order to study the direct influence of melatonin during the implantation period [55]. It has been shown that the differential expression of MT1 and MT2 receptors in pregnant and non-pregnant human uteri is able to influence the cyclic rhythm of myo-endometrial contractility [41].

Studies performed in a mouse culture system displayed that melatonin may be involved in metabolism to promote both the quality and the quantity of embryo development, and that it stimulates the formation of blastocysts during embryogenesis [56].

In vivo studies in mice have reported the ability of melatonin treatment to increase estradiol levels (E2), favoring implantation, but reducing the uterine receptivity period [57]. Similarly, treatment has improved the uterine microenvironment, promoting antioxidant enzymes expression, such as SOD, and catalase (CAT) [58].

From a molecular point of view, melatonin regulates the functionality of p53 by inducing p38-dependent phosphorylation of p53 itself [59]. It was then found that the p53-dependent DNA damage response activation is mediated by MT1 and MT2 in mice [60]. Furthermore, recent research has also shown that MT2 is more up-regulated, highlighting its dominant role in mediating the melatonin action in reproduction. The p21 up-regulation increased p38 activation, favoring p53 phosphorylation and activation, together with its up-regulation [61] (Figure 1). These results indicate that p53 might be a downstream element of MT1-MT2 activation, lastly regulating LIF expression, thereby influencing positively the embryo implantation [62].

Considering that during the pre-implantation period, oxidative stress negatively influences embryo quality and pregnancy success [63], these observations led to the use of melatonin as an adjuvant in in vitro embryo cultures, increasing the embryonic development and blastocyst rate in mice [64].

**Figure 2:**
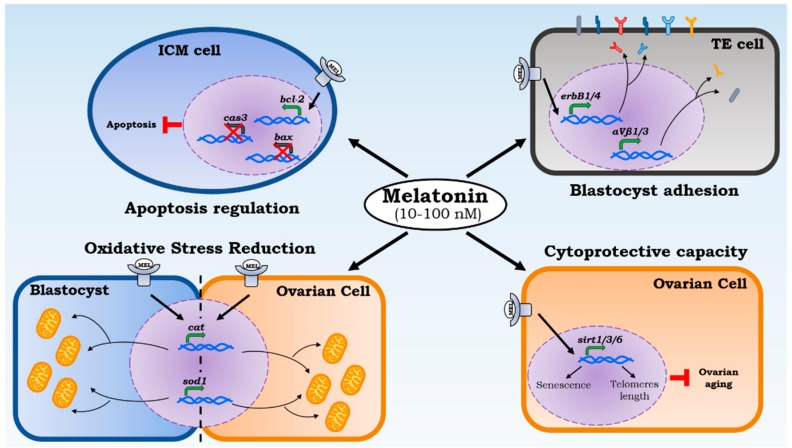
Melatonin local effects on implantation. Melatonin acts on different levels of implantation capacity. The cytoprotective capacity allows us to check ovarian aging by increasing sirtuins transcription (Sirt1/3/6) that protect the 3′ end of chromosomes (telomeres) reducing the cellular senescence. ROS reduction is led by melatonin’s direct action and biosynthesis of catalase and superoxide dismutase 1. Their transcription is powered through melatonin administration in ovarian cells and blastocysts. Moreover, the apoptosis regulation and a powered adhesion proteins expression by melatonin increase the rate of blastocyst implantation.

In this regard, blastocysts treated with melatonin display a significantly higher number of ICM and TE cells, improving intracellular ROS levels [65]. During in vitro culture, embryos are exposed to higher levels of oxidative stress than in vivo [66]. The cause of the increased susceptibility to oxidative stress of mammalian embryos is a high concentration of lipids [67]. Therefore, ROS increase causes loss of membrane integrity and alteration in functional structures [68]. Melatonin improves embryo quality through increasing expression levels of antioxidant genes, reducing mitochondrial damage and apoptosis. Studies on rats confirm melatonin’s ability to improve levels of catalase (CAT) and superoxide dismutase (SOD) if exposed to oxidative stress induced by sodium fluoride (NaF), counteracting ROS activity on embryos [69] (Figure 2). In addition, several studies have reported that melatonin is able to regulate apoptotic processes. In particular, according to the concentration of melatonin used, either this molecule can up-regulate the anti-apoptotic gene *bcl-2* and down-regulate pro-apoptotic *bax* and *caspase-3* genes [70], reducing apoptosis of embryonic cells and positively impacting mouse embryo cleavage rates (with concentrations of melatonin of 10 and 100 nM), or, in high concentrations (100 μM), can mediate pro-oxidant and pro-apoptotic actions, causing a delay in embryonic development and implantation speed (Figure 2). This last pro-oxidant activity was also reported in the study of Buyukavci et al., where the counteracting melatonin activity on leukemia cell growth is displayed [71]. For this reason, the therapeutic use of melatonin is promising; however, its dosage, especially in pregnancy, requires specific attentions and considerations.

The cytoprotective capacity of melatonin at the ovarian district can delay the aging effects and age-related diseases [72]. This ability of melatonin was confirmed by a recent study that has displayed how the telomere reduction was decreased with melatonin administration thanks to sirtuin 1 (SIRT1) up-regulation [73]. At the molecular level, ROS influences both the telomere length, protective structures located at the 3′ end of chromosomes [74], and the sirtuin activity [75]. The sirtuin family plays different roles in cell differentiation, senescence, and apoptosis regulation [76]. The sirtuins expressed in the ovary (SIRT1, SIRT3 and SIRT6) are markers of ovarian aging, as they are positively correlated with the follicular reserve [77] (Figure 2).

Indeed, the SIRT1 activity regulates the Eukaryotic Initiation Factor 2 (eIF2) phosphorylation [78], which has been found to be inactive in patients with neurodegenerative diseases, such as Parkinson’s and Alzheimer’s disease [79].

A further confirmation of the importance of melatonin for the development of the embryo is related to its gene expression activity. In some studies, the increase of the blastocyst quality was reflected in a significant increase in pregnancy rates and observed birth rates [80]. Promoting TE and ICM cells proliferation has yielded a positive effect on cleavage rates. In fact, TE cell reduction causes embryo mortality and implant failure [81], while a low number of ICM cells displays a higher probability of fetal loss or damage in development [82]. The success of embryonic implantation depends on the epidermal receptor expression of ErbB growth factors (ErbB1, ErbB2, ErbB3, ErbB4) in the blastocyst trophectoderm, and on their interaction with the ligands present on the receptive endometrial cells. *ErbB1* and *erbB4* are the first genes expressed on pre-implant blastocyst [83]. Several studies display an increase in the gene expression of *erbB1*, *pra*, *p53,* and *mt2* in mice treated with melatonin [84]. The signaling pathway begins from the heparin binding epidermal growth factor (HB-EGF), an early molecular marker, synthesized in the uterus, and adherent to blastocysts through ErbB1/4.

In vitro embryo production alters the expression of these receptors, as they are subjected to environmental stress [85]. Pre-treatment with melatonin in mice subjected to In Vitro Fertilization and Embryo Transfer (IVFET) highlighted the increase of HB-EGF expression in the endometrium [86], increasing the implantation probability [84]. Thus, melatonin administration, reducing oxidative stress, promotes the up-regulation of this factor, favoring the implantation [87].

Clinically, melatonin supplementation was added in IVF protocols, and the effects of this intervention were investigated by clinical trials, some of them randomized and controlled. Considering the proven positive effects of the supplementation of myo-inositol plus folic acid on the follicular fluid composition, and consequently on the oocyte quality, Rizzo et al. examined whether the addition of melatonin supplemented continuously from the day of GnRH administration further improved IVF outcomes. Results showed that the supplementation of melatonin is effective in ameliorating the activity of myo-inositol by improving both oocyte quality and pregnancy outcome in patients with low oocyte quality history [88]. Similarly, Unfer et al. demonstrated that the treatment with myo-inositol and melatonin improves ovarian stimulation protocols and pregnancy outcomes in infertile women with poor oocyte quality [89]. Furthermore, the addition of melatonin to myo-inositol and folic acid significantly improved both oocytes and embryo quality in Poly-cystic Ovary Syndrome (PCOS) women who underwent IVF procedures, thus highlighting a synergistic action between the two substances [90].

Overall, both experimental and clinical studies support the positive effects of melatonin on oocyte quality and embryo implantation, and patients with subfertility or infertility may benefit form melatonin supplementation. Therefore, despite the fact that additional randomized control trials (RCTs) are necessary to recommend the use of melatonin as a first line treatment for patients with poor fertility, clinicians should consider its supplementation as a safe and effective approach to increasing the chance of a successful pregnancy.

## 4. Melatonin Functions on Pregnancy Outcomes

The pineal gland, a site of melatonin synthesis and secretion, becomes mature after birth. Therefore, assuming the role of melatonin in pregnancy, it is evident that a strong connection exists between maternal secretion and normal fetal and embryonic development.

Melatonin treatment has been studied in different conditions and during the gestational period. Several studies have shown that melatonin administration from the first IVFET cycles, continued during pregnancy, has been associated with an improvement in pregnancy results [88].

As mentioned previously, melatonin can pass all biological barriers, including the placental one [15]. The passage of maternal melatonin exposes the fetus to a daily rhythm, with low concentrations during the day and high ones during the night, establishing the circadian rhythm [91]. The circadian rhythm is also induced in the fetal organs [92]. This is evidenced through the fetal heart frequency, which is synchronized with maternal activity (i.e., day-night), together with hormonal secretion and physique temperature rhythms [93]. These rhythms are interrupted if the mothers are kept in a continuous luminous environment until the end of the gestation period.

In vivo studies, in which mice were exposed to continuous light during the second half of gestation, contributed different results. First, there was delayed intrauterine growth, and second, the clock genes and steroidogenic genes expression was down-regulated in fetal adrenal, lowering corticosterone production. In this context, the importance of melatonin is evident, considering that with a daily dose administration of this molecule to the mother, a reverse of these alterations is achieved [94].

At the human fetal level, melatonin receptors are expressed in the central nervous system (CNS) and in several areas of the fetal brain [95]. At the molecular level, the binding of melatonin to the MT1 receptor [96] modulates the self-regulating interaction of clock genes, including *bmal-1*, *per1-3*, *cry1-2*, and *clock*, and their gene products [97]. These participate in circadian rhythm generation and maintenance through transcriptional feedback circuits [98]. The *clock* gene, constitutively expressed in the suprachiasmatic nucleus (SCN), and *bmal-1* gene, expressed cyclically, activates *per1-3* and *cry1-2* genes transcription. These accumulate in the nucleus, establishing complexes where, by negative feedback, they regulate *clock* and *bmal-1* genes transcription [99].

Melatonin receptors are also present in human peripheral tissues, highlighting how melatonin can also perform other functions [100]. Recent research shows that melatonin stimulates fetal adrenal growth [96], but inhibits cortisol synthesis in adrenal gland [101]. Furthermore, melatonin plays an essential role in influencing fetal gonads [102] and post-natal reproductive development [103]. Moreover, in the trophoblastic cells that constitute the placenta, the “clock genes” are expressed, and control the activated transcriptional feedback. The breaking of this coordinated process can compromise the protective function, with a consequent chain-reaction effect to the immune system [104].

Reporting the melatonin activity on placental tissue can be very useful in understanding the importance of this molecule for the fetus. Placental tissue is characterized by a very strong relationship between its two components, cytotrophoblast and syncytiotrophoblast, and plays a key role during implantation and throughout pregnancy. Recently, several studies reported melatonin activity at local levels and its production by the cytotrophoblast [105]. The role of melatonin at this level was also confirmed by the presence of melatonin membrane receptors both in the cytotrophoblast and in the syncytiotrophoblast. All these findings allowed us to formulate the hypothesis of paracrine and autocrine actions [106]. It is already known that melatonin can induce or block the apoptosis (see above) in cancer cells [107]. A balance between the formation of the syncytiotrophoblast from cytotrophoblasts and its degeneration via apoptosis is necessary to prevent pathologies from developing in the placenta. Melatonin, which, as pointed about above, is produced in cytotrophoblasts [105,106], has a prominent regulatory effect on apoptosis. It has been repeatedly demonstrated that melatonin exhibits anti-apoptotic actions in normal cells, while being pro-apoptotic in cancerous cells [107]. These dual functions are believed to be exploited by the placenta to maintain a balance between the villous cytotrophoblasts and the syncytiotrophoblast [108].

Considering that melatonin receptors are present at the fetal level, there is the evidence that melatonin is involved in neurodevelopment, i.e., developing fetal sleep patterns [109]. This displays how the action of melatonin on the human fetus is not limited to maintaining the circadian rhythm. In fact, during the last trimester of pregnancy, a more rapid development of the fetal brain is closely associated with Rapid Eye Movement (REM) sleep [110]. Melatonin might be one of the factors that regulates both the fetal sleep cycle REM and non-REM sleep [111].

The neuroprotective activity of melatonin, both in the fetal brain and in the adult, is fundamental for optimal development. This effect, and its effectiveness, have been reported in some studies on several animal models, in which the melatonin administration to the mother caused a significative reduction of fetal hypoxia, improving neurodevelopment and decreasing of brain lesions and oxidative stress in the fetus [112].

Tamura et al. proposed that deficient pineal melatonin production may be casually related to the development of spontaneous abortion in cases where chromosomal anomalies and/or uterine abnormalities have been excluded. Recurrent pregnancy loss has been associated with oxidative damage and immunological imbalance; in this context, melatonin acts as a direct powerful free-radical scavenger with immunomodulatory effects. Furthermore, melatonin stimulates the secretion of progesterone, and inhibits the synthesis of prostaglandins which are potent inducers of miscarriage and preterm labor [113].

An emerging concept that links environmental conditions during embryonic development with the risk of developing pathologies after birth is fetal programming. As seen previously, the expression of melatonin varies according to the several moments of fetal development, with fluctuations throughout the pregnancy. Modifications of this program, with altered serum levels, have been observed in complicated pregnancies, supporting the hypothesis of an influence on fetal development [114]. These modifications could be a response to environmental conditions, and increase the risk of the development of pathologies in adulthood [115]. At this regard, altered melatonin serum level seems to be related to anomalous placental development. The reason for this is unbalanced epigenetic mechanisms [116] induced by an increased oxidative stress, which is in turn linked with a higher formation of ROS and free radicals. This leads to incorrec methylation, an epigenetic alteration that could induce modifications in the gene expression patterns and phenotype modifications in adulthood [117].

Melatonin could regulate epigenetic mechanisms by nuclear receptors, leading to DNA bending in the oocyte [118]. This hypothesis suggests that melatonin could activate antioxidant enzyme expression by epigenetic mechanisms. Among these enzymes, Nrf2 seems play a crucial role in epigenetic modifications in fetal programming [119]. For this reason, correct melatonin levels during pregnancy are crucial to prevent fetal reprogramming, protecting the fetus from the risk of developing metabolic disease in adulthood

Hobson et al. have evaluated the impact of melatonin on women with severe preeclampsia; the obtained data have reported the ability of melatonin to improve maternal endothelial pro-oxidant injury, showing a very safe profile for mothers and their fetuses. Preeclampsia is a major disorder in human pregnancy, which may include potential fetal and maternal complications such as low birth weight, prematurity, and renal failure, Hemolysis, Elevated Liver enzymes, Low Platelet count (HELLP) syndrome, liver failure, and cerebral edema. The pathogenesis of preeclampsia is complex, and involves high placental oxidative stress [120]. A further confirmation that low night-time melatonin levels, as recorded in pregnant women with preeclampsia, resulted significantly lower than normal pregnancies [121]. So, melatonin supplementation should be considered as a potential disease-preventing agent, with the aim of extending pregnancy duration to improve clinical outcomes [120].

Throughout gestation, serum melatonin concentrations in the mother display fluctuations in both physiological and pathological pregnancies [122]. In particular, a significant increase in serum melatonin occurs after 24 weeks from implantation, increasing again after 32 weeks. Finally, after birth, the values return to physiological levels within two days [123]. This expression variability could be explained by the important role played by melatonin as a pregnancy regulators.

Although the mechanism that determines childbirth is not yet fully understood, a seasonal and daily periodicity of human birth has been identified [124]. The photoperiod would seem to be an important factor in controlling birth time. According to this logic, melatonin is the main mediator of this control, as reported [125]. Several studies have displayed an increase in melatonin levels in amniotic fluid during the pre-natal period. The changes in circulating melatonin levels would appear to be a determining factor in the birth times [126].

## 5. Conclusions

A large amount of research has focused on melatonin, shedding light on its mechanisms of action, and its functions. From these studies has emerged the key role of melatonin in processes of embryonic development and human reproduction.

Among the physiological activities of this molecule, one of the most important is the modulator of the circadian reproductive maternal rhythm, favoring uterine receptivity, and establishing the day/night cycle in the fetus.

Another fundamental activity of melatonin is the antioxidant function that allows the direct removal of free radicals from the oocyte and the embryo, supporting implantation and proliferation. More and more data confirm the cytoprotective effect, together with the immunomodulatory one, that would seem essential for the success of pregnancy and correct fetal development. Finally, the latest studies attest to a probable protective effect and in the direct development of the nervous system.

These results are validated by the fact that in models with alterations and/or reductions in melatonin secretion levels, efficiently desynchronizing the circadian rhythm, there is a higher probability of significant complications in pregnancy, such as abortion, pre-eclampsia, and neonatal neurological disability [127]. In fact, several data show that pregnant women who are often exposed to light during the night, or who perform night work, have increased incidences of complications during pregnancy. These complications might be related to a deficiency of melatonin secretion in these individuals [113].

Currently, melatonin is present as a food supplement and over-the-counter drug, with high availability, and has no acute or chronic adverse effects.

So far, several clinical studies have been performed on pregnant women: melatonin supplementation has shown no risk, but rather, has displayed positive effects. Therefore, all the obtained data confirm the important role of this molecule in supporting physiological pregnancy. In this regard, further studies would be useful to better define the posology of melatonin in order to achieve the most effective responses, both for the mother and the fetus.

## Abbreviations

CAT	Catalase
CL	Corpus luteum
CNS	central nervous system
E2	Oestradiol
GnRH	Gonadotropin-releasing hormone
GSH	Glutathione
HB-EGF	Heparin binding epidermal growth factor
ICAM-I	Intracellular adhesion molecule
ICM	Internal cell mass
LH	Luteinizing hormone
MUC1	Mucin
NaF	Sodium fluoride
P4	Progesterone
ROS	Reactive oxygen species
SIRT	Sirtuin
SOD	Superoxide dismutase
TE	Trophectoderm

## References

[1] Reiter R.J. (1993). The melatonin rhythm: Both a clock and a calendar. Experientia.

[2] Reiter R.J. (1998). Melatonin and human reproduction. Ann. Med..

[3] Mahal H.S., Sharma H.S., Mukherjee T. (1999). Antioxidant properties of melatonin: A pulse radiolysis study. Free Radic. Biol. Med..

[4] Garcia J.J., Lopez-Pingarron L., Almeida-Souza P., Tres A., Escudero P., Garcia-Gil F.A., Tan D.X., Reiter R.J., Ramirez J.M., Bernal-Perez M. (2014). Protective effects of melatonin in reducing oxidative stress and in preserving the fluidity of biological membranes: A review. J. Pineal Res..

[5] Ramis M.R., Esteban S., Miralles A., Tan D.X., Reiter R.J. (2015). Protective effects of melatonin and mitochondria-targeted antioxidants against oxidative stress: A review. Curr. Med. Chem..

[6] Barrett P., Bolborea M. (2012). Molecular pathways involved in seasonal body weight and reproductive responses governed by melatonin. J. Pineal Res..

[7] Dijk D.J., Duffy J.F. (1999). Circadian regulation of human sleep and age-related changes in its timing, consolidation and eeg characteristics. Ann. Med..

[8] Van Someren E.J. (2000). Circadian rhythms and sleep in human aging. Chronobiol. Int..

[9] Copinschi G., Van Cauter E. (1995). Effects of ageing on modulation of hormonal secretions by sleep and circadian rhythmicity. Horm. Res..

[10] Valenzuela F.J., Torres-Farfan C., Richter H.G., Mendez N., Campino C., Torrealba F., Valenzuela G.J., Seron-Ferre M. (2008). Clock gene expression in adult primate suprachiasmatic nuclei and adrenal: Is the adrenal a peripheral clock responsive to melatonin?. Endocrinology.

[11] Silver A.C., Arjona A., Walker W.E., Fikrig E. (2012). The circadian clock controls toll-like receptor 9-mediated innate and adaptive immunity. Immunity.

[12] Reiter R.J., Tamura H., Tan D.X., Xu X.Y. (2014). Melatonin and the circadian system: Contributions to successful female reproduction. Fertil. Steril..

[13] Summa K.C., Vitaterna M.H., Turek F.W. (2012). Environmental perturbation of the circadian clock disrupts pregnancy in the mouse. PLoS ONE.

[14] Reiter R.J., Tan D.X., Korkmaz A., Rosales-Corral S.A. (2014). Melatonin and stable circadian rhythms optimize maternal, placental and fetal physiology. Hum. Reprod. Update.

[15] Okatani Y., Okamoto K., Hayashi K., Wakatsuki A., Tamura S., Sagara Y. (1998). Maternal-fetal transfer of melatonin in pregnant women near term. J. Pineal Res..

[16] Lahiri D.K., Ge Y.W., Sharman E.H., Bondy S.C. (2004). Age-related changes in serum melatonin in mice: Higher levels of combined melatonin and 6-hydroxymelatonin sulfate in the cerebral cortex than serum, heart, liver and kidney tissues. J. Pineal Res..

[17] Handyside A.H., Montag M., Magli M.C., Repping S., Harper J., Schmutzler A., Vesela K., Gianaroli L., Geraedts J. (2012). Multiple meiotic errors caused by predivision of chromatids in women of advanced maternal age undergoing in vitro fertilisation. Eur. J. Hum. Genet..

[18] Li Q., Geng X., Zheng W., Tang J., Xu B., Shi Q. (2012). Current understanding of ovarian aging. Sci. China Life Sci..

[19] Grondahl M.L., Yding Andersen C., Bogstad J., Nielsen F.C., Meinertz H., Borup R. (2010). Gene expression profiles of single human mature oocytes in relation to age. Hum. Reprod..

[20] Jasmin, Peters V.M., Spray D.C., Mendez-Otero R. (2016). Effect of mesenchymal stem cells and mouse embryonic fibroblasts on the development of preimplantation mouse embryos. In Vitro Cell Dev. Biol. Anim..

[21] Moniruzzaman M., Hasan K.N., Maitra S.K. (2016). Melatonin actions on ovaprim (synthetic gnrh and domperidone)-induced oocyte maturation in carp. Reproduction.

[22] Guan S., Xie L., Ma T., Lv D., Jing W., Tian X., Song Y., Liu Z., Xiao X., Liu G. (2017). Effects of melatonin on early pregnancy in mouse: Involving the regulation of star, cyp11a1, and ihh expression. Int. J. Mol. Sci..

[23] Paria B.C., Reese J., Das S.K., Dey S.K. (2002). Deciphering the cross-talk of implantation: Advances and challenges. Science.

[24] Bergh P.A., Navot D. (1992). The impact of embryonic development and endometrial maturity on the timing of implantation. Fertil. Steril..

[25] Hertig A.T., Rock J., Adams E.C. (1956). A description of 34 human ova within the first 17 days of development. Am. J. Anat..

[26] Psychoyos A. (1986). Uterine receptivity for nidation. Ann. N. Y. Acad. Sci..

[27] Levine A.J., Tomasini R., McKeon F.D., Mak T.W., Melino G. (2011). The p53 family: Guardians of maternal reproduction. Nat. Rev. Mol. Cell Biol..

[28] Aplin J.D. (2000). The cell biological basis of human implantation. Baillieres Best Pract. Res. Clin. Obstet. Gynaecol..

[29] Aplin J.D., Hey N.A., Graham R.A. (1998). Human endometrial muc1 carries keratan sulfate: Characteristic glycoforms in the luminal epithelium at receptivity. Glycobiology.

[30] Hoffman L.H., Olson G.E., Carson D.D., Chilton B.S. (1998). Progesterone and implanting blastocysts regulate muc1 expression in rabbit uterine epithelium. Endocrinology.

[31] Zhou J., Qu C., Sun Q., Wu L., Liu Y., Yang Z., Zhang J. (2014). Sophoricoside fails the embryo implantation by compromising the uterine endometrial receptivity at implantation “window” of pregnant mice. Chem. Biol. Interact..

[32] Kimber S.J., Glasser S.R., Mulholland J., Psychoyos A. (1994). Carbohydrates and implantation of the mammalian embryo. Endocrinology of Embryo-Endometrium Interactions.

[33] Tabibzadeh S., Babaknia A. (1995). The signals and molecular pathways involved in implantation, a symbiotic interaction between blastocyst and endometrium involving adhesion and tissue invasion. Hum. Reprod..

[34] Campbell S., Swann H.R., Seif M.W., Kimber S.J., Aplin J.D. (1995). Cell adhesion molecules on the oocyte and preimplantation human embryo. Hum. Reprod..

[35] Schultz J.F., Mayernik L., Rout U.K., Armant D.R. (1997). Integrin trafficking regulates adhesion to fibronectin during differentiation of mouse peri-implantation blastocysts. Dev. Genet..

[36] Sharkey A.M., Smith S.K. (2003). The endometrium as a cause of implantation failure. Best Pract. Res. Clin. Obstet. Gynaecol..

[37] De Mouzon J., Rossin-Amar B., Bachelot A., Renon C., Devecchi A. (1998). Fivnat. Influence of attempt rank in in vitro fertilization. Contracept. Fertil. Sex..

[38] Favetta L.A., St John E.J., King W.A., Betts D.H. (2007). High levels of p66shc and intracellular ros in permanently arrested early embryos. Free Radic. Biol. Med..

[39] Lysiak J.J., Zheng S., Woodson R., Turner T.T. (2007). Caspase-9-dependent pathway to murine germ cell apoptosis: Mediation by oxidative stress, bax, and caspase 2. Cell Tissue Res..

[40] Roy D., Belsham D.D. (2002). Melatonin receptor activation regulates gnrh gene expression and secretion in gt1-7 gnrh neurons. Signal transduction mechanisms. J. Biol. Chem..

[41] Morgan M.A., Silavin S.L., Wentworth R.A., Figueroa J.P., Honnebier B.O., Fishburne J.I., Nathanielsz P.W. (1992). Different patterns of myometrial activity and 24-h rhythms in myometrial contractility in the gravid baboon during the second half of pregnancy. Biol. Reprod..

[42] Jang H., Lee O.H., Lee Y., Yoon H., Chang E.M., Park M., Lee J.W., Hong K., Kim J.O., Kim N.K. (2016). Melatonin prevents cisplatin-induced primordial follicle loss via suppression of pten/akt/foxo3a pathway activation in the mouse ovary. J. Pineal Res..

[43] Jang H., Na Y., Hong K., Lee S., Moon S., Cho M., Park M., Lee O.H., Chang E.M., Lee D.R. (2017). Synergistic effect of melatonin and ghrelin in preventing cisplatin-induced ovarian damage via regulation of foxo3a phosphorylation and binding to the p27(kip1) promoter in primordial follicles. J. Pineal Res..

[44] Brannstrom M., Norman R.J. (1993). Involvement of leukocytes and cytokines in the ovulatory process and corpus luteum function. Hum. Reprod..

[45] Ronnberg L., Kauppila A., Leppaluoto J., Martikainen H., Vakkuri O. (1990). Circadian and seasonal variation in human preovulatory follicular fluid melatonin concentration. J. Clin. Endocrinol. MeTable.

[46] Tamura H., Takasaki A., Miwa I., Taniguchi K., Maekawa R., Asada H., Taketani T., Matsuoka A., Yamagata Y., Shimamura K. (2008). Oxidative stress impairs oocyte quality and melatonin protects oocytes from free radical damage and improves fertilization rate. J. Pineal Res..

[47] Salhab M., Dhorne-Pollet S., Auclair S., Guyader-Joly C., Brisard D., Dalbies-Tran R., Dupont J., Ponsart C., Mermillod P., Uzbekova S. (2013). In vitro maturation of oocytes alters gene expression and signaling pathways in bovine cumulus cells. Mol. Reprod. Dev..

[48] Adriaens I., Jacquet P., Cortvrindt R., Janssen K., Smitz J. (2006). Melatonin has dose-dependent effects on folliculogenesis, oocyte maturation capacity and steroidogenesis. Toxicology.

[49] Bronson F.H. (1995). Seasonal variation in human reproduction: Environmental factors. Q. Rev. Biol..

[50] Partonen T. (1999). Short note: Melatonin-dependent infertility. Med. Hypotheses.

[51] Takahashi M. (2012). Oxidative stress and redox regulation on in vitro development of mammalian embryos. J. Reprod. Dev..

[52] Gao C., Han H.B., Tian X.Z., Tan D.X., Wang L., Zhou G.B., Zhu S.E., Liu G.S. (2012). Melatonin promotes embryonic development and reduces reactive oxygen species in vitrified mouse 2-cell embryos. J. Pineal Res..

[53] Chuffa L.G., Seiva F.R., Favaro W.J., Teixeira G.R., Amorim J.P., Mendes L.O., Fioruci B.A., Pinheiro P.F., Fernandes A.A., Franci J.A. (2011). Melatonin reduces lh, 17 beta-estradiol and induces differential regulation of sex steroid receptors in reproductive tissues during rat ovulation. Reprod. Biol. Endocrinol..

[54] Reiter R.J., Mayo J.C., Tan D.X., Sainz R.M., Alatorre-Jimenez M., Qin L. (2016). Melatonin as an antioxidant: Under promises but over delivers. J. Pineal Res..

[55] Zhang L., Zhang Z., Wang F., Tian X., Ji P., Liu G. (2017). Effects of melatonin administration on embryo implantation and offspring growth in mice under different schedules of photoperiodic exposure. Reprod. Biol. Endocrinol..

[56] Asgari Z., Ghasemian F., Ramezani M., Bahadori M.H. (2012). The effect of melatonin on the developmental potential and implantation rate of mouse embryos. Cell J..

[57] Ma W.G., Song H., Das S.K., Paria B.C., Dey S.K. (2003). Estrogen is a critical determinant that specifies the duration of the window of uterine receptivity for implantation. Proc. Natl. Acad. Sci. USA.

[58] Richter H.G., Hansell J.A., Raut S., Giussani D.A. (2009). Melatonin improves placental efficiency and birth weight and increases the placental expression of antioxidant enzymes in undernourished pregnancy. J. Pineal Res..

[59] Mediavilla M.D., Cos S., Sanchez-Barcelo E.J. (1999). Melatonin increases p53 and p21waf1 expression in mcf-7 human breast cancer cells in vitro. Life Sci..

[60] Santoro R., Mori F., Marani M., Grasso G., Cambria M.A., Blandino G., Muti P., Strano S. (2013). Blockage of melatonin receptors impairs p53-mediated prevention of DNA damage accumulation. Carcinogenesis.

[61] Proietti S., Cucina A., Dobrowolny G., D’Anselmi F., Dinicola S., Masiello M.G., Pasqualato A., Palombo A., Morini V., Reiter R.J. (2014). Melatonin down-regulates mdm2 gene expression and enhances p53 acetylation in mcf-7 cells. J. Pineal Res..

[62] Zhao J., Fu B., Peng W., Mao T., Wu H., Zhang Y. (2017). Melatonin protect the development of preimplantation mouse embryos from sodium fluoride-induced oxidative injury. Environ. Toxicol. Pharmacol..

[63] Wang H., Dey S.K. (2006). Roadmap to embryo implantation: Clues from mouse models. Nat. Rev. Genet..

[64] Ishizuka B., Kuribayashi Y., Murai K., Amemiya A., Itoh M.T. (2000). The effect of melatonin on in vitro fertilization and embryo development in mice. J. Pineal Res..

[65] Moshkdanian G., Moghani-Ghoroghi F., Pasbakhsh P., Nematollahi-Mahani S.N., Najafi A., Kashani S.R. (2017). Melatonin upregulates erbb1 and erbb4, two primary implantation receptors, in pre-implantation mouse embryos. Iran. J. Basic. Med. Sci..

[66] Choi J., Park S.M., Lee E., Kim J.H., Jeong Y.I., Lee J.Y., Park S.W., Kim H.S., Hossein M.S., Jeong Y.W. (2008). Anti-apoptotic effect of melatonin on preimplantation development of porcine parthenogenetic embryos. Mol. Reprod. Dev..

[67] Sturmey R.G., Reis A., Leese H.J., McEvoy T.G. (2009). Role of fatty acids in energy provision during oocyte maturation and early embryo development. Reprod. Domest. Anim..

[68] Tamura H., Takasaki A., Taketani T., Tanabe M., Kizuka F., Lee L., Tamura I., Maekawa R., Aasada H., Yamagata Y. (2012). The role of melatonin as an antioxidant in the follicle. J. Ovarian Res..

[69] Bharti V.K., Srivastava R.S., Kumar H., Bag S., Majumdar A.C., Singh G., Pandi-Perumal S.R., Brown G.M. (2014). Effects of melatonin and epiphyseal proteins on fluoride-induced adverse changes in antioxidant status of heart, liver, and kidney of rats. Adv. Pharmacol. Sci..

[70] Mohseni M., Mihandoost E., Shirazi A., Sepehrizadeh Z., Bazzaz J.T., Ghazi-khansari M. (2012). Melatonin may play a role in modulation of bax and bcl-2 expression levels to protect rat peripheral blood lymphocytes from gamma irradiation-induced apoptosis. Mutat. Res..

[71] Buyukavci M., Ozdemir O., Buck S., Stout M., Ravindranath Y., Savasan S. (2006). Melatonin cytotoxicity in human leukemia cells: Relation with its pro-oxidant effect. Fundam. Clin. Pharmacol..

[72] Martin M., Macias M., Escames G., Leon J., Acuna-Castroviejo D. (2000). Melatonin but not vitamins c and e maintains glutathione homeostasis in t-butyl hydroperoxide-induced mitochondrial oxidative stress. FASEB J..

[73] Hardeland R. (2013). Melatonin and the theories of aging: A critical appraisal of melatonin’s role in antiaging mechanisms. J. Pineal Res..

[74] Ozturk S., Sozen B., Demir N. (2014). Telomere length and telomerase activity during oocyte maturation and early embryo development in mammalian species. Mol. Hum. Reprod..

[75] Tatone C., Di Emidio G., Vitti M., Di Carlo M., Santini S., D’Alessandro A.M., Falone S., Amicarelli F. (2015). Sirtuin functions in female fertility: Possible role in oxidative stress and aging. Oxid. Med. Cell Longev..

[76] Watroba M., Szukiewicz D. (2016). The role of sirtuins in aging and age-related diseases. Adv. Med. Sci..

[77] Zhang J., Fang L., Lu Z., Xiong J., Wu M., Shi L., Luo A., Wang S. (2016). Are sirtuins markers of ovarian aging?. Gene.

[78] Ghosh H.S., Reizis B., Robbins P.D. (2011). Sirt1 associates with eif2-alpha and regulates the cellular stress response. Sci. Rep..

[79] Chang R.C., Yu M.S., Lai C.S. (2006). Significance of molecular signaling for protein translation control in neurodegenerative diseases. Neurosignals.

[80] Tian X., Wang F., Zhang L., Ji P., Wang J., Lv D., Li G., Chai M., Lian Z., Liu G. (2017). Melatonin promotes the in vitro development of microinjected pronuclear mouse embryos via its anti-oxidative and anti-apoptotic effects. Int. J. Mol. Sci..

[81] Kelly S.M., Robaire B., Hales B.F. (1992). Paternal cyclophosphamide treatment causes postimplantation loss via inner cell mass-specific cell death. Teratology.

[82] Shiao N.H., Chan W.H. (2009). Injury effects of ginkgolide b on maturation of mouse oocytes, fertilization, and fetal development in vitro and in vivo. Toxicol. Lett..

[83] Jeong W., Jung S., Bazer F.W., Song G., Kim J. (2016). Epidermal growth factor: Porcine uterine luminal epithelial cell migratory signal during the peri-implantation period of pregnancy. Mol. Cell Endocrinol..

[84] He C., Wang J., Li Y., Zhu K., Xu Z., Song Y., Song Y., Liu G. (2015). Melatonin-related genes expressed in the mouse uterus during early gestation promote embryo implantation. J. Pineal Res..

[85] Wang F., Tian X., Zhou Y., Tan D., Zhu S., Dai Y., Liu G. (2014). Melatonin improves the quality of in vitro produced (ivp) bovine embryos: Implications for blastocyst development, cryotolerance, and modifications of relevant gene expression. PLoS ONE.

[86] Moghani-Ghoroghi F., Moshkdanian G., Sehat M., Nematollahi-Mahani S.N., Ragerdi-Kashani I., Pasbakhsh P. (2018). Melatonin pretreated blastocysts along with calcitonin administration improved implantation by upregulation of heparin binding-epidermal growth factor expression in murine endometrium. Cell J..

[87] Wang F., Tian X., Zhang L., Tan D., Reiter R.J., Liu G. (2013). Melatonin promotes the in vitro development of pronuclear embryos and increases the efficiency of blastocyst implantation in murine. J. Pineal Res..

[88] Rizzo P., Raffone E., Benedetto V. (2010). Effect of the treatment with myo-inositol plus folic acid plus melatonin in comparison with a treatment with myo-inositol plus folic acid on oocyte quality and pregnancy outcome in ivf cycles. A prospective, clinical trial. Eur. Rev. Med. Pharmacol. Sci..

[89] Unfer V., Raffone E., Rizzo P., Buffo S. (2011). Effect of a supplementation with myo-inositol plus melatonin on oocyte quality in women who failed to conceive in previous in vitro fertilization cycles for poor oocyte quality: A prospective, longitudinal, cohort study. Gynecol. Endocrinol..

[90] Pacchiarotti A., Carlomagno G., Antonini G., Pacchiarotti A. (2016). Effect of myo-inositol and melatonin versus myo-inositol, in a randomized controlled trial, for improving in vitro fertilization of patients with polycystic ovarian syndrome. Gynecol. Endocrinol..

[91] Seron-Ferre M., Torres-Farfan C., Forcelledo M.L., Valenzuela G.J. (2001). The development of circadian rhythms in the fetus and neonate. Semin. Perinatol..

[92] Kivela A., Kauppila A., Leppaluoto J., Vakkuri O. (1989). Serum and amniotic fluid melatonin during human labor. J. Clin. Endocrinol. MeTable.

[93] Mirmiran M., Maas Y.G., Ariagno R.L. (2003). Development of fetal and neonatal sleep and circadian rhythms. Sleep Med. Rev..

[94] Mendez N., Abarzua-Catalan L., Vilches N., Galdames H.A., Spichiger C., Richter H.G., Valenzuela G.J., Seron-Ferre M., Torres-Farfan C. (2012). Timed maternal melatonin treatment reverses circadian disruption of the fetal adrenal clock imposed by exposure to constant light. PLoS ONE.

[95] Thomas L., Purvis C.C., Drew J.E., Abramovich D.R., Williams L.M. (2002). Melatonin receptors in human fetal brain: 2-[(125)i]iodomelatonin binding and mt1 gene expression. J. Pineal Res..

[96] Torres-Farfan C., Rocco V., Monso C., Valenzuela F.J., Campino C., Germain A., Torrealba F., Valenzuela G.J., Seron-Ferre M. (2006). Maternal melatonin effects on clock gene expression in a nonhuman primate fetus. Endocrinology.

[97] Hawkins G.A., Meyers D.A., Bleecker E.R., Pack A.I. (2008). Identification of coding polymorphisms in human circadian rhythm genes per1, per2, per3, clock, arntl, cry1, cry2 and timeless in a multi-ethnic screening panel. DNA Seq..

[98] Hastings M.H. (2000). Circadian clockwork: Two loops are better than one. Nat. Rev. Neurosci..

[99] Reppert S.M., Weaver D.R. (2002). Coordination of circadian timing in mammals. Nature.

[100] Drew J.E., Williams L.M., Hannah L.T., Barrett P., Abramovich D.R. (1998). Melatonin receptors in the human fetal kidney: 2-[125i]iodomelatonin binding sites correlated with expression of mel1a and mel1b receptor genes. J. Endocrinol..

[101] Torres-Farfan C., Richter H.G., Germain A.M., Valenzuela G.J., Campino C., Rojas-Garcia P., Forcelledo M.L., Torrealba F., Seron-Ferre M. (2004). Maternal melatonin selectively inhibits cortisol production in the primate fetal adrenal gland. J. Physiol..

[102] Gunduz B., Stetson M.H. (1994). Effects of photoperiod, pinealectomy, and melatonin implants on testicular development in juvenile siberian hamsters (phodopus sungorus). Biol. Reprod..

[103] Shaw D., Goldman B.D. (2007). Developmental changes in male siberian hamsters (phodopus sungorus) exposed to different gestational and postnatal photoperiods. J. Pineal Res..

[104] Waddell B.J., Wharfe M.D., Crew R.C., Mark P.J. (2012). A rhythmic placenta? Circadian variation, clock genes and placental function. Placenta.

[105] Iwasaki S., Nakazawa K., Sakai J., Kometani K., Iwashita M., Yoshimura Y., Maruyama T. (2005). Melatonin as a local regulator of human placental function. J. Pineal Res..

[106] Lanoix D., Beghdadi H., Lafond J., Vaillancourt C. (2008). Human placental trophoblasts synthesize melatonin and express its receptors. J. Pineal Res..

[107] Sainz R.M., Mayo J.C., Rodriguez C., Tan D.X., Lopez-Burillo S., Reiter R.J. (2003). Melatonin and cell death: Differential actions on apoptosis in normal and cancer cells. Cell Mol. Life Sci..

[108] Lanoix D., Lacasse A.A., Reiter R.J., Vaillancourt C. (2012). Melatonin: The smart killer: The human trophoblast as a model. Mol. Cell Endocrinol..

[109] Gitto E., Marseglia L., Manti S., D’Angelo G., Barberi I., Salpietro C., Reiter R.J. (2013). Protective role of melatonin in neonatal diseases. Oxid. Med. Cell Longev..

[110] Morrissey M.J., Duntley S.P., Anch A.M., Nonneman R. (2004). Active sleep and its role in the prevention of apoptosis in the developing brain. Med. Hypotheses.

[111] Cajochen C., Krauchi K., Mori D., Graw P., Wirz-Justice A. (1997). Melatonin and s-20098 increase rem sleep and wake-up propensity without modifying nrem sleep homeostasis. Am. J. Physiol..

[112] Supramaniam V.G., Jenkin G., Loose J., Wallace E.M., Miller S.L. (2006). Chronic fetal hypoxia increases activin a concentrations in the late-pregnant sheep. BJOG.

[113] Tamura H., Nakamura Y., Terron M.P., Flores L.J., Manchester L.C., Tan D.X., Sugino N., Reiter R.J. (2008). Melatonin and pregnancy in the human. Reprod. Toxicol..

[114] Tamura H., Kawamoto M., Sato S., Tamura I., Maekawa R., Taketani T., Aasada H., Takaki E., Nakai A., Reiter R.J. (2017). Long-term melatonin treatment delays ovarian aging. J. Pineal Res..

[115] Barker D.J., Winter P.D., Osmond C., Margetts B., Simmonds S.J. (1989). Weight in infancy and death from ischaemic heart disease. Lancet.

[116] Ribatti D., Nico B., Crivellato E. (2009). Morphological and molecular aspects of physiological vascular morphogenesis. Angiogenesis.

[117] Korkmaz A., Sanchez-Barcelo E.J., Tan D.X., Reiter R.J. (2009). Role of melatonin in the epigenetic regulation of breast cancer. Breast Cancer Res. Treat..

[118] Irmak M.K., Topal T., Oter S. (2005). Melatonin seems to be a mediator that transfers the environmental stimuli to oocytes for inheritance of adaptive changes through epigenetic inheritance system. Med. Hypotheses.

[119] Korkmaz A., Rosales-Corral S., Reiter R.J. (2012). Gene regulation by melatonin linked to epigenetic phenomena. Gene.

[120] Hobson S.R., Gurusinghe S., Lim R., Alers N.O., Miller S.L., Kingdom J.C., Wallace E.M. (2018). Melatonin improves endothelial function in vitro and prolongs pregnancy in women with early-onset preeclampsia. J. Pineal Res..

[121] Bouchlariotou S., Liakopoulos V., Giannopoulou M., Arampatzis S., Eleftheriadis T., Mertens P.R., Zintzaras E., Messinis I.E., Stefanidis I. (2014). Melatonin secretion is impaired in women with preeclampsia and an abnormal circadian blood pressure rhythm. Ren. Fail..

[122] Kivela A. (1991). Serum melatonin during human pregnancy. Acta Endocrinol..

[123] Nakamura Y., Tamura H., Kashida S., Takayama H., Yamagata Y., Karube A., Sugino N., Kato H. (2001). Changes of serum melatonin level and its relationship to feto-placental unit during pregnancy. J. Pineal Res..

[124] Anderka M., Declercq E.R., Smith W. (2000). A time to be born. Am. J. Public Health.

[125] Tamura H., Takayama H., Nakamura Y., Reiter R.J., Sugino N. (2008). Fetal/placental regulation of maternal melatonin in rats. J. Pineal Res..

[126] Mitchell M.D., Sayers L., Keirse M.J., Anderson A.B., Turnbull A.C. (1978). Melatonin in amniotic fluid during human parturition. Br. J. Obstet. Gynaecol..

[127] Man G.C.W., Zhang T., Chen X., Wang J., Wu F., Liu Y., Wang C.C., Cheong Y., Li T.C. (2017). The regulations and role of circadian clock and melatonin in uterine receptivity and pregnancy-an immunological perspective. Am. J. Reprod. Immunol..

